# Photodynamic Therapy for Peri-Implant Diseases

**DOI:** 10.3390/antibiotics11070918

**Published:** 2022-07-08

**Authors:** Betul Rahman, Anirudh Balakrishna Acharya, Ruqaiyyah Siddiqui, Elise Verron, Zahi Badran

**Affiliations:** 1 Periodontology Unit, Department of Preventive and Restorative Dentistry, College of Dental Medicine, University of Sharjah, Sharjah P.O. Box 27272, United Arab Emirates; brahman@sharjah.ac.ae (B.R.); aacharya@sharjah.ac.ae (A.B.A.); 2 College of Arts and Sciences, University City, American University of Sharjah, Sharjah P.O. Box 26666, United Arab Emirates; ruqaiyyahsiddiqui35@gmail.com; 3 CNRS, UMR 6230, CEISAM, UFR Sciences et Techniques, Université de Nantes, 2, rue de la Houssinière, BP 92208, CEDEX 3, 44322 Nantes, France; elise.verron@univ-nantes.fr

**Keywords:** peri-implantitis, photodynamic therapy, photosensitizers, microorganisms

## Abstract

Peri-implant diseases are frequently presented in patients with dental implants. This category of inflammatory infections includes peri-implant mucositis and peri-implantitis that are primarily caused by the oral bacteria that colonize the implant and the supporting soft and hard tissues. Other factors also contribute to the pathogenesis of peri-implant diseases. Based on established microbial etiology, mechanical debridement has been the standard management approach for peri-implant diseases. To enhance the improvement of therapeutic outcomes, adjunctive treatment in the form of antibiotics, probiotics, lasers, etc. have been reported in the literature. Recently, the use of photodynamic therapy (PDT)/antimicrobial photodynamic therapy (aPDT) centered on the premise that a photoactive substance offers benefits in the resolution of peri-implant diseases has gained attention. Herein, the reported role of PDT in peri-implant diseases, as well as existing observations and opinions regarding PDT, are discussed.

## 1. Introduction

Dental implants are a mainstay in oral rehabilitation for replacing lost teeth and improving the quality of life in people who have such therapeutic needs. The long-term functional survival rate of dental implants has been established [1,2,3,4,5,6,7,8]. However, success, though clinically commendable, is not the same as long-term survival owing to complications [9]. Dental implants must be maintained by the individual patient, including professional assistance for durable function.

Maintenance entails optimal oral hygiene with periodic professional interventions to ensure the health of the peri-implant tissues and the sustainable status of the dental implant. If maintenance protocols are not complied with on a regular basis, peri-implant diseases could ensue as a complication. Peri-implant diseases differ from periodontal disease [10] and have a prevalence reaching 43% according to certain reports [11,12,13]. It is to be noted that the factors contributing to peri-implant diseases, other than those associated with the patient, are related to the clinician, the site and design of the implant and the type of prosthesis.

An association between poor oral biofilm (dental plaque) control and peri-implant disease has been reported, underpinning the periodontal microbiome as a primary etiologic agent [13,14]. A quantitative growth of the oral biofilm increased the odds of developing peri-implant diseases, implying that dental implant patients who do not maintain proper oral hygiene are almost four times more likely to be afflicted with peri-implant diseases [11,15]. Needless to state, a combined maintenance effort by the dental implant patient (home oral hygiene care) and the clinician (professionally administered procedures) will decrease the occurrence of peri-implant diseases [16,17]. Although these practices employing stringent home care for oral biofilm control and professional mechanical debridement and adjunctive therapies may have led to a decrease in the pathological microbial burden, they have not led to a thorough resolution from a clinical point of view [17,18,19].

Hence, the challenge of preventing peri-implant diseases has seen a continuous quest for exploring feasible therapeutic modalities. One of these avenues is the search for a clinically beneficial adjunctive remedy to bolster the resolution of peri-implant diseases. Adjunctive therapies include antibiotics, antiseptics, probiotics, air abrasives, lasers and photodynamic therapy (PDT). This narrative review seeks to appraise the potential value of PDT in peri-implant diseases.

## 2. Peri-Implant Diseases

Peri-implant diseases are pathologic inflammatory conditions that include peri-implant mucositis and peri-implantitis. Similar to gingivitis being a precursor to periodontitis, peri-implant mucositis (reversible) is likewise to peri-implantitis (irreversible). Peri-implant mucositis and peri-implantitis are primarily caused by the oral biofilm [20], with other contributing risk factors such as genetics, systemic diseases (ex., diabetes mellitus), tobacco abuse, anatomical features such as inadequate width of the keratinized gingiva, prosthetic design, occlusal overload and patient-related issues of poor oral hygiene maintenance and lack of supportive professional treatment. Moreover, the cause of peri-implant bone loss is attributed to metallosis, which ascribes the release of titanium particles and ions as an inflammatory response to the oral biofilm, or by tribocorrosion/fretting, resulting in corrosion of the implant surface [21]. Evidence exists for the presence of metal particles in soft tissues around titanium implants [22]. 

The case definitions and classification of peri-implant mucositis and peri-implantitis are recognized [23]:

The definition of peri-implant mucositis is based on the existence of peri-implant signs of inflammation (redness, swelling, bleeding on probing), with an absence of additional bone loss after initial healing. The definition of peri-implantitis is based on the presence of peri-implant signs of inflammation, radiographic evidence of bone loss after initial bone remodeling and increasing probing depth when compared with probing depth measurements obtained after the prosthesis placement. If previous radiographs are unavailable, radiographic bone level of more than or equal to 3 mm in combination with bleeding on probing and probing depths of more than or equal to 6 mm is indicative of peri-implantitis. It is reiterated that both peri-implant mucositis and peri-implantitis are oral biofilm-associated pathologic conditions [24]. The routine treatment modality in preventing and controlling these conditions is thorough mechanical removal of the deposits. This has led to the use of adjunctive therapy that includes antiseptics and systemic and local antibiotics to facilitate control of peri-implant biofilms. However, such additional measures have not been found to always significantly improve the treatment outcomes [25]. Antibiotics have been considered advantageous due to the relative simplicity in administration to the patient as an adjunct to mechanical debridement [26,27]. However, antibiotics generally have unwanted side effects, most importantly antibiotic resistance, and have not exhibited clinical improvements or microbiological resolution as compared to mechanical treatment alone [28,29]. Therefore, considering that the etiopathogenesis is primarily driven by the putative pathogens in the oral biofilm, an emphasis on alternative adjunctive therapy is being placed on PDT.

## 3. Photodynamic Therapy 

The effect of visible light on acridine hydrochloride in the killing of *Paramecia caudatum* was observed by Oscar Raab in Munich, Germany, more than a hundred and twenty years ago [30]. The essential involvement of light, a photosensitive agent and oxygen led to the coining of the word “photodynamic” by von Tappeiner in 1904 [31]. The leading-edge work by Wilson in 1993 [32] paved the way to establishing the plausible efficacy of PDT and its role as an alternative to antibiotics in the eliminating of oral biofilm pathogens.

PDT for use in humans is founded on the concept that, when a light-sensitive agent called a photosensitizer is selectively taken up by microorganisms, it will absorb light of specific wavelengths to be eventually activated in the presence of oxygen. This results in the production of singlet oxygen (^1^O_2_*) and free radicals that are lethal to microorganisms by way of being cytotoxic. The molecular nature of singlet oxygen potentially prevents development of resistance from the microorganisms [33]. The lifetime of the singlet oxygen is in nanoseconds that barely permits any interaction with other molecules in the surrounding regions [34,35]. This excited molecule may revert to the ground state or convert to a triplet state (lifetime is micro- to milliseconds) that may produce phosphorescence while returning to the ground state, or it can react in Type I and Type II photo-processes [36]. For simple clarity, Type I involves the release of free radicals such as superoxide, hydroxyl and lipid-derived radicals [37], and Type II produces excited-state singlet oxygen that oxidizes lipids, proteins and nucleic acids, causing cytotoxicity [38]. In PDT, singlet oxygen is the most damaging, having a 100 nm diffusion distance and less than 0.04 µs half-life [35,39,40]. PDT damages the cytoplasmic membrane, as well as the DNA of the microbiota [41,42].

### 3.1. Photosensitizers

**Figure 1:**
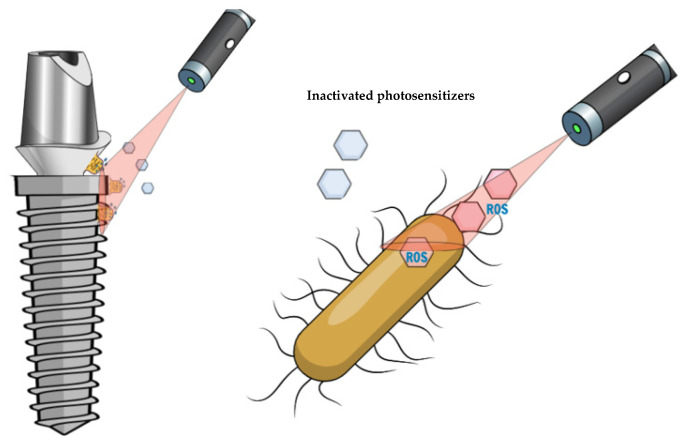
Antibacterial photodynamic therapy for implant decontamination. In peri-implantitis cases, bacterial biofilms develop on the prosthetic components and the exposed implant surfaces. Photosensitizers can bond to bacterial walls or penetrate bacterial cells before activation and cytotoxic ROS release. ROS: reactive oxygen species, released after laser activation of photosensitizers.

Photosensitizers absorb light of specific wavelengths, transforming it to energy. Dougherty and colleagues [43] introduced the first photosensitizer called “hematoporphyrin derivative” (HpD), which was later purified and came to be known as Photofrin. Many of the photosensitizers were developed for cancer therapy based on the tetrapyrrole nucleus, such as porphyrins, chlorins, bacteriochlorins and phthalocyanines [44]. Recently, synthetic dyes (phenothiazines (methylene blue and toluidine blue), rose bengal, squaraines, boron dipyrromethene (BODIPY) dyes, phenalenones, transition metal compounds), natural derivatives (hypericin, hypocrellin, riboflavin, curcumin, pterin, parietin, chlorin, 5-aminolevilunic acid) and nanoparticles have been used and researched [45,46]. The frequently used photosensitizers for oral use are phenothiazine chloride, phenothiazines (toluidine blue, methylene blue), aurogreen and indocyanine green. Methylene blue, for instance, has been in use for about a century; its low molecular weight, positive charge and hydrophilicity permit its crossing through the porin protein channels of the cell membrane of Gram-negative bacteria and its interaction with lipopolysaccharides [47,48,49,50]. Methylene blue shows maximum absorption of light wavelength 660 nm [51] and toluidine blue 630 nm [47] for killing microorganisms. In a nutshell, a photosensitizer that binds to microorganisms is activated by light of a suitable wavelength in the presence of oxygen, leading to the generation of reactive oxygen species (Figure 1) that are cytotoxic to the particular microorganisms, causing damage to the cytoplasmic membrane and DNA [52]. This is known as lethal photosensitization [53], and when PDT targets microorganisms, it is referred to as antimicrobial PDT (aPDT) [54] or photoantimicrobial chemotherapy (PACT). The response to PTD may be influenced by the concentration of the photosensitizer, subgingival environmental pH, the time of dye penetration pre-irradiation, existence of any exudates, the light source, the dose of energy and the fluence rate (energy delivered per unit area) applied [55,56,57].

### 3.2. Activators of Photosensitizers

It was demonstrated that photosensitizers could be activated by using a dental curing light with effective antimicrobial results [58,59]. Lasers are a better light source due to some of their unique characteristics, such as being monochromatic, coherent and collimated and having narrow bandwidth, controllable wavelength and high optical power for activating photosensitizers [60]. Diode lasers are the most preferred light activator of photosensitizers in oral PDT, owing to economic convenience and portability as compared with helium–neon, argon, gallium–aluminum–arsenic diode lasers, aluminum gallium indium phosphide, erbium-doped yttrium aluminum garnet (Er: YAG), neodymium-doped yttrium aluminum garnet (Nd: YAG) and chromium-doped yttrium scandium gallium garnet (Cr: YSGG), as evidenced in the literature [33,57,61]. The wavelength compatibility of diode lasers with the frequently used phenothiazine photosensitizers is another reason for their preference.

## 4. PDT for Peri-Implant Diseases

Elimination of or reduction in the oral biofilm remains the cornerstone for preventing and treating peri-implant diseases. As mentioned earlier, mechanical debridement is the most important therapeutic modality, but with its limitations, the search for improving treatment outcomes of peri-implant disease has made PDT of great interest as an adjunctive therapy.

The literature has a wide range of information and data about the role of PDT in peri-implant diseases that need to be reviewed for better perspective.

### 4.1. PDT and Implant Surfaces

Peri-implant diseases are initiated by polymicrobial colonization of the peri-implant tissues and implant surfaces [62]. It becomes imperative to decontaminate implant surfaces as part of treating peri-implant diseases. Investigations about implant surface decontamination have provided insights regarding the use of PDT.

At this juncture, it is important to outline the microbiota involved in peri-implant diseases. A systematic review and meta-analysis by Sahrmann et al. [63] concluded that there was an increased prevalence of *Aggregatibacter actinomycetemcomitans* (*A.a*) and *Prevotella intermedia* (*P.i*) in peri-implantitis biofilms compared with healthy implant sites. *Actinomyces* spp., *Porphyromonas* spp. and *Rothia* spp. were found in periodontal/peri-implant sites that were healthy and with periodontitis and peri-implantitis, implying an inconsistent microbial profile. Moreover, conflicting reports exist regarding the detection of putative pathogens in sites of peri-implant disease and periodontal/peri-implant sites of health [64,65]. In spite of such variation in information, it is known that the oral microbiota affects the electroconductive characteristics of titanium, leading to its corrosion [66]. *Streptococcus mutans (S. mutans)* has been implicated in titanium corrosion [67,68] and possible metallosis.

In a first-of-its-kind study, Cai et al. [69] incubated *Staphylococcus aureus* (*S. aureus)* biofilm on polished and sandblasted large-grit acid-etched (SLA) titanium surfaces for 48 h, which were then randomly grouped for treatment protocols with phosphate-buffered saline, 0.2% chlorhexidine (CHX), 3% hydrogen peroxide (H_2_O_2_), PDT, 0.2% CHX plus PDT, and 3% H_2_O_2_ plus PDT. Colony-forming units (CFUs) were estimated for antimicrobial effects. The *S. aureus* biofilm was assessed with scanning electron microscopy (SEM) and confocal laser scanning microscope (CLSM). Their results concluded that 0.2% CHX plus PDT was more effective in eradicating *S. aureus* when compared with either treatment alone, as was 3% H_2_O_2_ plus PDT. This is suggestive that PDT provides an added benefit. At this point, it is to be noted that surface roughness parameters of the implant contribute to biofilm formation, i.e., smoother surfaces inhibit biofilm formation, and yet wettability of the surface enhances biofilm formation [70,71,72]. Such aspects will pose challenges in drawing definitive conclusions regarding surface decontamination of implant surfaces irrespective of using PDT, although Cai et al. mentioned that surface roughness did not have a bearing on the decontamination protocols used in their investigation. Considering that the aforementioned study evaluated mono species, the efficacy of implant surface decontamination by PDT on multiple peri-implant pathogens needs to be viewed. A comparative study by Azizi et al. [73] using PDT plus toluidine blue, PDT plus phenothiazine chloride, light-activated disinfection (LAD) by light-emitting diodes (LED) plus toluidine blue, and toluidine blue only on titanium implant surfaces contaminated with *P.i*, *A.a* and *Porphyromonas gingivalis* (*P.g*) revealed that the PDT protocols were more effective when compared with LED plus toluidine blue and with toluidine blue alone on a three-day-old biofilm. The same group of investigators performed another study where zirconia implants were contaminated with *A.a, P.i* and *P.g* [74], using similar protocols that included PDT plus toluidine blue, PDT plus phenothiazine chloride, (LED) plus toluidine blue and toluidine blue without light. The results pointed out that PDT protocols and LAD showed high and equal effectiveness in decontamination of zirconia implant surfaces. This is possibly because bacterial attachment affinity to zirconia surfaces is less than that to titanium surfaces due to variability in surface free energy and surface roughness [75,76]. Although the literature indicates the efficacy of PDT in bacterial killing on titanium surfaces [73,77,78,79,80], it is well to note that the effect of PDT may be better on the relatively smoother surfaces of zirconia implants. PDT seemingly does not per se alter the surface of the implant [81]. Another observation of interest is that bacteria such as *P.g* may endogenously produce photosensitizers, thus influencing PDT [82]. The effects of PDT (indocyanine plus diode laser), Er:YAG laser, LED and toluidine blue O photosensitizer, and 0.2% CHX on the elimination of *A.a* on SLA implant surfaces were assessed. Photodynamic therapy and LED with photosensitizers were shown to suppress *A.a* more effectively than Er:YAG laser irradiation. Although all the techniques resulted in lowering the counts of *A.a*, CHX fared better than the other methods of decontamination [83]. This was in line with an earlier, similar study comparing CHX and PDT on nonspecific salivary bacterial contamination of titanium surfaces [79]. CHX exhibits attachment to the implant surface and substantivity with bactericidal action up to 24 h [54,84]. This property of CHX would have influenced the results, and it is to be borne in mind that the evaluation was mono species. However, CHX has shown to be toxic to host cells when compared with light-activated therapy [85,86].

A recent report [87] studied sterile implants and subgingival biofilm-contaminated implants brushed with sterile saline, brushed with sterile saline and subjected to air-powder abrasive system plus sodium bicarbonate, and brushed with sterile saline and subjected to PDT, proposing that the air-powder abrasive system plus sodium bicarbonate and PDT protocols were the most efficient for in vitro decontamination of titanium implant surfaces (double acid etching, cylindrical, external hexagon); PDT showed greater reduction in anaerobic/microaerophilic nonspecific microbial CFUs. From the point of view of advocacy for PDT vis-à-vis an air-powder abrasive system, alteration of the surface characteristics of the implant and the risk of emphysema are demerits of the latter [88,89,90,91].

PDT has also been compared with laser therapy alone. Low-level laser therapy (LLLT) and PDT were investigated in vitro [92] by using them on cultures of subgingival periodontal biofilm obtained from periodontitis patients mimicking peri-implantitis and stock cultures of *S. aureus*. The authors of this study claim reduction in CFUs by LLLT and PDT in both cultures, with PDT being more effective. However, these results may not be able to extrapolate to implant surfaces. Implant decontamination may alter the surface characteristics [93,94,95,96,97,98], for example, as mentioned earlier, the air-powder polishing system. However, when laser, PDT and CHX were tested on SLA titanium contaminated with *A.a*, SEM and energy-dispersive X-ray spectroscopy (EDS) demonstrated no alterations in the surface characteristics of the implant [99]. The adhesiveness of substances on biomaterials is an important therapeutic factor [100]. Another facet of PDT and implants is the retention of the photosensitizer on the implant surface. The fluidic nature of the photosensitizers make retention a challenge that may affect therapeutic success. Hence, the modification of photosensitizers with certain biopolymers (methylcellulose, chitosan) is gaining research momentum. For example, the effectiveness of a quaternary ammonium chitosan on the retention of methylene blue on biofilm-contaminated SLA titanium surface and the elimination of *A.a* and *S. mutans* has shown promising results [101]. Generally, some investigations have found similarities in microbiological profiles between healthy and contaminated implant surfaces [102,103,104], whereas others have reported a more complex microbiota on implant surfaces [105,106]. No studies have reported an association of PDT and metallosis related to peri-implantitis.

### 4.2. Evaluation of PDT

The value of any therapeutic procedure lies in the tangible and beneficial clinical outcomes. Most studies involving the clinical efficacy of PDT on peri-implant diseases have considered the changes in parameters, such as probing pocket depth, clinical attachment loss, plaque and bleeding indices, and microbiological and radiographic assessments. As PDT primarily effects the microbiota, some relevant information about the same will be presented first in this section.

Several reports (involving a few to many bacterial species) reveal the peri-implant pathogens to be *A.a*, *P.g.*, *P.i.*, *Treponema denticola* (*T.d.*)*, Tannerella forsythia* (*T.f.*), *Fusobacterium nucleatum* (*F.n.*), *Campylobacter rectus* (*C.r.*), *Eikenella corrodens* (*E.c.*), *Peptostreptococcus micros* (*P.s.*) and others [107,108,109,110,111,112,113,114]. It seems reasonable to accept that peri-implant diseases, especially peri-implantitis, predominantly harbor *A.a*, *P.g.* and *P.i.*, although other putative species have also been detected [115]. A recent systematic review [116] concluded that PDT lowers the numbers of peri-implant pathogens *A.a*, *P.g.*, *P.i.*, *T.d.*, *F.n.* and *C.r.* The systematic review also doubted the benefit of Er:YAG laser in PDT.

The photosensitizer is the key in PDT, i.e., it needs to selectively penetrate the bacterial cell wall and should not be toxic to the host cells [117], and PDT is effective in inactivating Gram-positive bacteria due to the structural composition of the cell wall when compared with Gram-negative bacteria [118]. Therefore, the killing of Gram-positive bacteria by PDT is possibly better achieved as compared with Gram-negative bacteria [115,119,120]. This aspect has an impact on the acceptance of PDT’s efficacy in peri-implant disease control. However, experimental findings show that *P.g.* and *A.a.* are susceptible to PDT [121,122].

The second consideration in this section is a comparison of PDT with antibiotics used as adjunctive therapy. Regarding periodontitis, if the use of antibiotics as an adjunct needs justification, Maan et al. [123] in their systematic review concluded that better clinical outcomes are observed, supported by another systematic review and meta-analysis by Zhao et al. [124], who compared systemic antibiotics and PDT as adjuncts in periodontitis and peri-implantitis. Another review [125] contradicts such observations by stating that both systemic antibiotics and PDT as adjuncts in periodontitis (it did not include peri-implantitis) were not convincing in obtaining clinical improvements in probing depths, clinical attachment levels and bleeding on probing. The systematic review by Øen et al. in 2021 [126] opined that adjunctive systemic antibiotics cannot be considered as standard treatment in peri-implantitis. This is, again, not in line with Zhao et al. [124].

The local delivery of antibiotics and PDT have also been explored. A comparison between minocycline microspheres and PDT in peri-implantitis has shown comparable improvements suggestive of PDT as an alternative [110,127], and locally delivered metronidazole compared with PDT in smokers with peri-implantitis exhibited equal benefits in clinical, microbiological and immunological outcomes [128]. This may have an implication to the conclusion of Javed et al., who stated that the use of both systemic and local antibiotics in peri-implantitis is debatable [129].

Both systemic and local antibiotics do not have a beneficial role in peri-implant mucositis as per Jepsen et al. [13], but PDT may have short-term influence in controlling inflammation of both peri-implant mucositis and peri-implantitis, as inferred by Sculean et al. [130].

### 4.3. PDT and Modifying/Risk Factors

Genetics, history of periodontitis, iatrogenic factors, tobacco abuse and uncontrolled diabetes mellitus are some of the modifying/risk factors; genetic polymorphisms/past history of periodontitis, excess cement at the implant site, tobacco use and diabetes mellitus are associated with peri-implant diseases [131,132,133]. A brief focused examination of tobacco use and diabetes mellitus in relation to PDT follows. Sgolastra et al. refute tobacco smoking as a risk factor for peri-implantitis [134]. However, the failure of implant osseointegration is high, and the rate of failure of implants is double in tobacco smokers as compared with nonsmokers and may be a predictor of implant failure [135,136,137,138]. Findings from a longitudinal study [139] show improvement in parameters such as plaque index, bleeding on probing, probing depth and bone loss when mechanical debridement with adjunctive PDT was used to treat peri-implantitis in water-pipe users (“hookah”/“shisha”) and tobacco smokers. Similar improvements were noted in peri-implant mucositis treated with a combination of mechanical debridement and PDT versus mechanical debridement alone in a group of smokeless tobacco users [140]. However, PDT plus mechanical debridement failed to reduce a large number of subgingival microbial species in another report [141]. Despite some investigations contradicting each other about the influence of diabetes mellitus on peri-implantitis and showing inability to establish a definitive association [142,143,144], the emphasis on diabetes mellitus as a risk for peri-implantitis has been recognized [145,146]. The efficacy of PDT in improving clinical peri-implant disease parameters, pro-inflammatory biomarkers and microbiological profiles has been reported [132,147,148,149]. It is interesting that one investigation [150] involving pre-diabetes and smokers who were treated for peri-implant mucositis concluded that PDT and mechanical debridement were compromised in pre-diabetes (both smokers and nonsmokers) but effective in the non-diabetic group (both smokers and nonsmokers). It is challenging to draw a firm conclusion based on the reports in the literature about the efficacy of PDT as an adjunct in peri-implant disease patients who have modifying/risk factors such as tobacco usage and diabetes mellitus. However, possible benefits of adjunctive PDT in such conditions can be anticipated from a clinical perspective.

### 4.4. Randomized Controlled Trials of PDT as an Adjunct to Mechanical Debridement

Several randomized controlled trials [110,127,151,152,153,154,155,156,157,158,159,160,161] of PDT as adjunctive therapy for peri-implant diseases have evaluated its efficacy, with some reporting improvement (in parameters such as plaque scores, bleeding on probing, probing depths, mucosal recession, clinical attachment levels, crestal bone loss by radiographic assessments, counts of putative microbes) and some not in agreement. These trials included the comparison of PDT with mechanical debridement (some with air-powder abrasive systems, local antibiotics, open flap debridement) with mostly the use of diode lasers, LED and phenothiazine photosensitizers. The follow-up period generally ranged from 3 months (or less) to 1 year. The clinical outcome parameters (other than microbiological parameters in some) in a majority of these trials were probing depths, plaque and bleeding indices, bleeding on probing, clinical attachment loss/recession and radiographic assessments.

Two trials concluded that PDT plus mechanical debridement is as effective as local antibiotic delivery plus mechanical debridement [110,127]. Romeo et al. [151] stated the use of PDT as a reliable co-adjuvant to mechanical debridement (inclusive of surgical intervention) and graft placement. However, Alharthi et al. [152], based on their results, reflected that adjunctive PDT is helpful in alleviating peri-implant mucositis but does not contribute to osseous regeneration. Deeb et al. [153] found additional benefits of adjunctive PDT regarding bleeding scores in tobacco smokers with peri-implant disease. Javed et al. [154] and Rifaiy et al. [155] reported better efficacy of PDT plus mechanical debridement in tobacco smokers and e-cigarette (vaping) users, respectively. Some investigators were convinced that PDT as an adjunct to mechanical debridement was valuable in the treatment of peri-implant diseases [156,157,158]. Table 1 summarizes a selection of the randomized controlled trials that show PDT to be potentially beneficial in the treatment of peri-implant diseases. However, De Angelis et al. [159], Esposito et al. [160] and Albaker et al. [161] did not find any clinical outcome improvements employing adjunctive PDT.

## 5. Critical Overview

Thus far, this review has presented information about the role of PDT in peri-implant diseases. The literature seemingly has supported the adjunctive use of PDT, with some results in contradiction. To provide a standpoint from the highest level of evidence, a network meta-analysis of randomized controlled trials of PDT as adjunctive therapy for peri-implantitis definitively concluded in favor of adjunctive PDT in comparison with other interventions, such as mechanical debridement alone or mechanical debridement combined with local drug delivery [162].

Table 2 shows the other relevant systematic reviews with or without meta-analyses [124,163,164,165,166,167,168,169,170,171,172] in the past recent years that outline the role of PDT in peri-implant disease treatment with inconclusive, tentative or definitive conclusions. Four of these systematic reviews and meta-analyses are inconclusive; two affirm the role of PDT in bacterial load reduction with another proposing PDT as an alternative to antibiotics; one review has a tentative conclusion; two categorically deny PDT to have added benefits; and another review suggests mechanical debridement alone is better (though a combination therapy with adjuncts may be beneficial). While PDT is used complying to safety standards, one concern is the toxicity of photosensitizers, and the other is the harmful irradiation (of lasers) to the eyes of the patient and the clinical personnel involved during the procedure [173,174].

However, Alqutub [175] concluded that in the short term, PDT as an adjunct to mechanical debridement is useful in peri-implant soft tissue diseases. A recent overview of systematic reviews and meta-analyses in 2022 by Joshi et al. [176] is indicative of PDT to be effective therapy for peri-implant diseases, although the availability of long-term data is a concern.

Deliberating on the entirety of adjunctive PDT as a treatment option for peri-implant diseases, the question remains as to whether it can be an absolutely reliable and useful procedure ensuring predictable and beneficial clinical outcomes.

## 6. Conclusions

To conclude emphatically about the role of PDT in peri-implant diseases may be difficult due to varying study designs and data sets. From an objective point of view, the inference of this review is that PDT reduces bacterial load related to peri-implant diseases and may be considered as an alternative to antibiotics. PDT seemingly offers short-term benefits as an adjunct to mechanical debridement in the treatment of peri-implant diseases, as indicated by the majority of the randomized controlled trials reviewed. However, as with most treatment procedures, PDT for peri-implant diseases needs judicious case selection and administration in clinical situations, for example, after specific microbial identification. Interpretation of this review’s relevance and findings for clinical practice should be weighed and executed on a customized basis for individual patients, and future studies are warranted to determine the unequivocal role of PDT in peri-implant diseases.

## Figures and Tables

**Table 1:** Summary of Randomized Controlled Trials of PDT as an adjunctive therapy in peri-implant diseases.

Author(s)	Year	Study Type	Comparison	Study Population	Outcome Measures	Follow-up Period	Results	Conclusions
Bassetti et al. [110]	2014	RCT	LDD vs. PDT	Initial PerImp	BOP, PD, CAL, REC, RBL, BLd	12 months	Improvement in parameters	Both the therapies are effective; PDT may be used as an alternative to LDD
Schar et al. [127]	2013	RCT	LDD vs. PDT	Initial PerImp	BOP, PD, CAL, REC, Pl (modified)	6 months	Significant changes in BOP, PD, REC, PI (modified) in both groups	Both the therapies are effective; PDT may be used as an alternative to LDD
Romeo et al. [151]	2016	RCT	MD vs. PDT	PerImp	PI, BOP, PD	6 months	Improvement in PI, BOP, PD	PDT is a reliable adjunct
Al Harthi et al. [152]	2022	RCT	MD vs. MD+PDT at different time periods	PerImp	PI, GI, PD, RBL	9 months	Significant improvements in parameters using MD+PDT compared with MD	PDT as an adjunct is effective in resolving PerImM
Deeb et al. [153]	2020	RCT	MD vs. MD+PDT vs. MD+SysAB in cigarette smokers	PerImp	BOP, PI, PD, BLd	3 months	Improved parameters in combination therapy groups	PDT is comparable to systemic antibiotics as adjunct to MD
Javed et al. [154]	2017	RCT	MD vs. MD+PDT in cigarette smokers	PerImM	BOP, PI, PD	3 months	PI and PD improved but no significant change in BOP	MD+PDT is better than MD alone in cigarette smokers
Karimi et al. [156]	2016	RCT	MD vs. MD+PDT	PerImp	BOP, GI, PD, CAL	3 months	Improved PD and CAL in MD+PDT	MD+PDT is beneficial
Rakašević et al. [157]	2016	RCT	PDT vs. CHX	PerImp	BOP, PI, PD, BLd	3 months	Improved BOP and BLd in PDT group	PDT may be used as adjuvant in implant surface decontamination
Wang et al. [158]	2019	RCT	MD vs. PDT	PerImp	BOP, PI, PD, CAL	6 months	Improved parameters in PDT group	MD+PDT is better than MD

RCT: Randomized Controlled Trial; vs.: Versus; PDT: Photodynamic Therapy; MD: Mechanical Debridement; SysAB: Systemic Antibiotics; CHX: Chlorhexidine; PerImp: Peri-implantitis; BOP: Bleeding on Probing; RBL: Radiographic Bone Loss; PD: Probing Depth; REC: Mucosal Recession; CAL: Clinical Attachment Level; LDD: Local Drug Delivery; PerImM: Peri-implant Mucositis; PI: Plaque Index; GI: Gingival Index; BLd: Bacterial Load.

**Table 2:** Summary of systematic reviews and meta-analyses of PDT as an adjunctive therapy in peri-implant diseases.

Author(s)	Year	Study Type	Comparison	Study Population	Outcome Measures	Follow-Up Period	Results	Conclusions
Kotsakis et al. [163]	2014	SR+MA	LT/PDT longitudinal	PerImp	PD, CAL	6 months	Er:YAG and diode laser effective with phenothiazine photosensitizer; limited data regarding CO_2_ laser	Inconclusive due to heterogeneity of methodology
Faggion et al. [164]	2014	SR+MA	PDT and others vs. MD	PerImp	PD	?	MD+antibiotics achieved maximum PD reduction	Inconclusive
Chambrone et al. [165]	2018	SR+MA	PDT+ MD vs. MD	CP, AgP, PerImp	CAL, PD	˃3 months	Significant but modest differences between groups	PDT may provide similar clinical improvements as compared with conventional treatment
Albaker et al. [166]	2018	SR	PDT/LT vs. MD	PerImM	BOP, PD, PI	3–34 months	Significant improvement in parameters in all studies assessed	Inconclusive due to heterogeneity of methodology
Fraga et al. [167]	2018	SR+MA	Only PDT longitudinally	PerImp	BLd	?	Significant reduction in *A.a*., *P.g.*, *P.i*. counts	PDT effective in bacterial load reduction
Shiau [168]	2019	SR+MA	PDT and MD	PerImp	?	?	No clinical significance	PDT does not provide additional benefit
Lopez et al. [169]	2020	SR	Only PDT longitudinally	PD, PerImp	BOP, PD, CAL, PI, GI, BLd	3 months(?)	Improvements in all parameters	Significant reduction in bacterial load
Saneja et al. [170]	2020	SR+MA	LT/PDT longitudinal	PerImp, PerImM	PD, CAL	6–12 months	No significant results	LT/PDT has no superior efficacy (better in PerImM)
Zhao et al. [124]	2021	SR+MA	PDT vs. antibiotics	PD, PerImp	PD, CAL, BOP	3 months	Equal significance of PDT and antibiotics	PDT may be an alternative to antibiotics
Francis et al. [171]	2022	SR	PDT and others	In vitro on Titanium	Implant surface	?	MD is better; diode more effective than other lasers	MD better; combination procedures may provide improved results
Shahmohammadi et al. [172]	2022	SR+MA	PDT+MD vs. MD	Smokers with PerImp	BOP, PD	6 months	Significant differences between groups	Inconclusive due to heterogeneity of methodology
Joshi et al. [176]	2022	SR+MA Overview	Comparison of SR+MA of different non-surgical therapies	PerImp	Clinical	Variable	Significant differences	PDT is beneficial

SR: Systematic Review; MA: Meta-analyses; vs.: Versus; PDT: Photodynamic Therapy; MD: Mechanical Debridement; PerImp: Peri-implantitis; BOP: Bleeding on Probing; PD: Probing Depth; CP: Chronic Periodontitis; AgP: Aggressive Periodontitis; CAL: Clinical Attachment Level; LT: Laser Therapy; PerImM: Peri-implant Mucositis; PI: Plaque Index; GI: Gingival Index; BLd: Bacterial Load; ?: Unspecified/unknown.
